# A Molecular Dynamics Simulation of Complexes of Fullerenes and Lysine-Based Peptide Dendrimers with and without Glycine Spacers

**DOI:** 10.3390/ijms25020691

**Published:** 2024-01-05

**Authors:** Valeriy V. Bezrodnyi, Sofia E. Mikhtaniuk, Oleg V. Shavykin, Nadezhda N. Sheveleva, Denis A. Markelov, Igor M. Neelov

**Affiliations:** 1 Department of Physics, St. Petersburg State University, 7/9 Universitetskaya Nab., 199034 St. Petersburg, Russia; v.bezrodniy@spbu.ru (V.V.B.); kupala-89@mail.ru (O.V.S.); n.n.sheveleva@spbu.ru (N.N.S.); d.markelov@spbu.ru (D.A.M.); 2 Center of Chemical Engineering (CCE), St. Petersburg National Research University of Information Technologies, Mechanics and Optics (ITMO University), Kronverkskiy pr. 49, 197101 St. Petersburg, Russia; mikhtanyuk@mail.ru; 3 Department of Mathematics, Tver State University, Sadoviy Per., 35, 170102 Tver, Russia; 4 Institute of Macromolecular Compounds RAS, Bolshoi Prospect 31, 199004 St. Petersburg, Russia

**Keywords:** peptide dendrimers, fullerenes C_60_ and C_70_, complexes, drug delivery

## Abstract

The development of new nanocontainers for hydrophobic drugs is one of the most important tasks of drug delivery. Dendrimers with hydrophobic interiors and soluble terminal groups have already been used as drug carriers. However, the most convenient candidates for this purpose are peptide dendrimers since their interiors could be modified by hydrophobic amino acid residues with a greater affinity for the transported molecules. The goal of this work is to perform the first molecular dynamics study of the complex formation of fullerenes C_60_ and C_70_ with Lys-2Gly, Lys G2, and Lys G3 peptide dendrimers in water. We carried out such simulations for six different systems and demonstrated that both fullerenes penetrate all these dendrimers and form stable complexes with them. The density and hydrophobicity inside the complex are greater than in dendrimers without fullerene, especially for complexes with Lys-2Gly dendrimers. It makes the internal regions of complexes less accessible to water and counterions and increases electrostatic and zeta potential compared to single dendrimers. The results for complexes based on Lys G2 and Lys G3 dendrimers are similar but less pronounced. Thus, all considered peptide dendrimers and especially the Lys-2Gly dendrimer could be used as nanocontainers for the delivery of fullerenes.

## 1. Introduction

Classical dendrimers are regular star-like (starburst) macromolecules that originate from a single core and branch in each generation. They have a well-defined molecular weight, nanoscale size, low polydispersity, and multivalency. Dendrimers can be used in many industrial, biomedical, and pharmaceutical applications due to their unique properties [1]. They are considered nanocontainers for drug and gene delivery since they can form complexes with different bioactive molecules [2]. The diversity of chemical structures of dendrimers and the possibility of their modification facilitates the development of delivery systems with desired properties for various drug molecules [3]. The formation of such complexes depends on the nature of the interaction between the dendrimer and guest molecules. This may be simple physical entrapment or encapsulation due to hydrophobic, electrostatic interactions, or hydrogen bonding [4]. The use of a dendrimer-based system can also help achieve controlled and targeted delivery to specific tissues and organs [5]. Dendrimer scaffolds protect the transported molecules from premature enzymatic degradation and early release and helps cross the biological membranes, improving their pharmacokinetics and bioavailability [6,7,8,9,10].

To achieve successful delivery and reduce adverse side effects, a reliable and safe delivery system should be non-cytotoxic, non-inflammatory, bioavailable, and biodegradable [11]. Polyamidoamine (PAMAM) and polypropyleneimine (PPI) dendrimers are well studied and well characterized as dendrimer-based delivery systems [12]. However, an important obstacle to the clinical use of these synthetic dendrimers is their toxicity. Various strategies have been developed to address the limitations due to the toxicity of dendrimer carriers [13,14]. For example, terminal groups of the dendrimers can be modified by PEGylation [15], acetylation [16], amino acids, or short peptides [17,18]. In this aspect, peptide dendrimers have some advantages because they consist of natural amino acid residues [19,20]. Poly-L-lysine (PLL) dendrimers are well-known representatives of peptide dendrimers. In recent years, drug formulations based on lysine dendrimers have been widely studied [21,22,23,24,25], and their safety and biodegradability have been demonstrated. It has been shown that peptide dendrimers themselves have antibacterial [26,27,28,29,30], antiviral [31,32], and antiangiogenic properties [33,34,35,36]. Moreover, the use of peptide dendrimers together with transported drug molecules can provide a synergistic effect of their action on cancer cells [37]. In addition, a peptide dendrimer can be synthesized with almost any desired set of amino acid residues [38,39,40,41,42] to improve the encapsulation of entrapped bioactive molecules.

The encapsulation ability of dendrimers helps overcome the difficulties associated with poor solubility and aggregation of guest molecules [25], allowing the use of smaller drug doses [43]. The possibility of delivering bioactive molecules using dendrimers was demonstrated, for example, for enoxaparin [44], berberine [45], quercetin [46], resveratrol [47], curcumin [48], anticancer drugs such as doxorubicin [49,50], and paclitaxel [51,52]. There were also many MD simulation works on the interaction of dendrimers with other bioactive molecules. See, for example, the experimental and simulation study of drug-loading into dendrimer [53], the MD study of acetyl-terminated PAMAM dendrimers for the pH-sensitive delivery of irinotecan and fluorouracil [54], MD simulations of carbon quantum dots PAMAM dendrimer nanocomposites [55], PAMAM dendrimers with G-actin [56], dendrimers with si-RNA [57], and cationic pyridylphenylene dendrimers with multicomponent anionic liposomes [58].

One of the bioactive molecules, whose biomedical applications are limited by its low water solubility, is fullerene [59]. Fullerene molecules are very attractive from a biological point of view due to their sizes of about 1 nm. However, they tend to form aggregates in water solutions. Aggregation leads to a fundamentally different interaction with biological structures due to the different sizes and properties of aggregates [60]. Since the discovery of fullerenes [61], opportunities for their use in biomedicine have been investigated [62,63]. Fullerenes have even been considered a promising drug delivery system; however, clinical trials have barely been started yet [63,64]. Fullerenes have antioxidative [65,66,67], antibacterial [68], antiviral [69], and antitumor [70,71,72] activities.

To improve the solubility of fullerene, the most commonly used method is to obtain its water-soluble derivatives via the attachment to many hydroxyl, carboxyl, or hydrophilic amino groups [73,74]. It was shown that the conjugation of fullerenes with cyclodextrins [75], peptides [76], and hydrophilic ethylene glycol spacers [77] significantly increases their solubility [78,79]. However, multiple functionalization of fullerenes decreases their bioactivity. The encapsulation of fullerenes into carbon nanotubes [80,81] and liposomes [82] has been proposed as a possible route for their delivery. In attempts to combine the beneficial properties of dendrimers and fullerenes, one resorted to the development of conjugates of fullerenes with dendrimers [83,84,85,86,87,88,89,90,91,92,93,94,95,96,97], as well as to the functionalization of dendrimers by fullerenes. Besides experimental work in this direction, there are several computer simulation works on dendrimer–fullerene conjugates [98,99,100].

However, investigations of guest–host complexes of fullerenes with dendrimers are poorly represented in the literature [101,102,103,104]. Perhaps this is due to the difficulties in obtaining the stable encapsulation of fullerenes into a dendrimer macromolecule. The purpose of this work is to study the possibility of creating complexes between a peptide dendrimer and fullerene. Since both of these nanosized molecules exhibit antitumor activity [37,105,106], we can assume that the strong synergetic effect [107] of their combination is possible. Another advantage of using peptide dendrimers is the ability to tune the interaction of the dendrimer with the guest molecule. This could be carried out not only by changing terminal groups but also by adding or changing amino acid residues in inner segments of peptide dendrimers. The use of different hydrophobicity of amino acid residues in the inner segments [108,109,110,111] could make it possible to achieve a suitable hydrophobic environment for the fullerene inside the dendrimer.

## 2. Results and Discussion

### 2.1. Formation of a Dendrimer–Fullerene Complex

**Figure 1:**
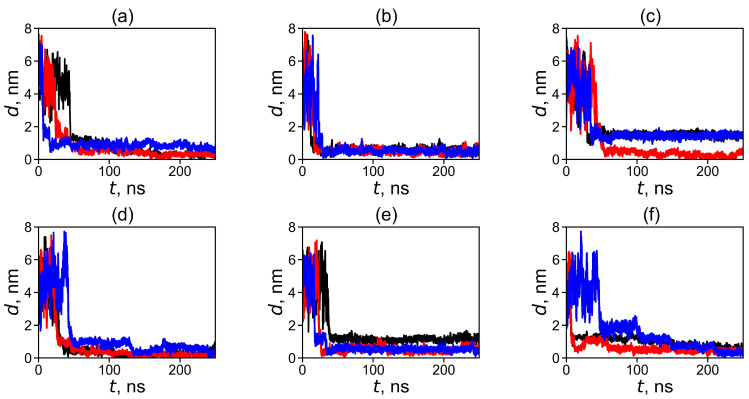
Distance between the centers of mass of the dendrimer and fullerene molecules in the system: (**a**) Lys-2Gly + C_60_, (**b**) Lys G2 + C_60_, (**c**) Lys G3 + C_60_, (**d**) Lys-2Gly + C_70_, (**e**) Lys G2 + C_70_, (**f**) Lys G3 + C_70_ at the first 250 ns of a trajectory (before and after the formation of complex). For each system, the three different initial conformations (positions of fullerene) were used and marked by different colors. The abrupt decrease in the distance reflects the formation of dendrimer–fullerene complexes.

In order to trace the formation of the dendrimer–fullerene complex and its stability, we calculated the time dependence of the distance, *d*(*t*), between the centers of mass of the dendrimer and fullerene molecules, as well as the time dependencies of the size of the subsystem consisting of the dendrimer and fullerene. The time dependencies of the distance between the dendrimers and fullerenes are shown in Figure 1. As can be seen from this figure, at the beginning of the simulation, the distances have large initial values of ~4–6 nm since the fullerene molecule was initially placed quite far from the center of mass of the dendrimer. At first, the *d*(*t*) curves fluctuate strongly for some time in all systems and then begin to decrease gradually due to intermolecular interactions between the dendrimer and the fullerene. Later, we can observe an abrupt step-like decrease in the value of *d*(*t*). After this, in most cases, the function *d*(*t*) practically does not change with time and fluctuates slightly near the average value close to 1 nm or less. This means that fullerenes are adsorbed on dendrimers, forming stable complexes with them. For some systems (Lys-2Gly + C_60_ (Figure 1a) and Lys G3 + C_70_ (Figure 1f)), the *d*(*t*) after the abrupt decrease continues to decrease slightly, reaching a constant value of *d* in the region 200–250 ns of the first half of total 500ns trajectory, which is used to establish equilibrium in the system.

Thus, we can conclude that in all the systems considered, a stable dendrimer–fullerene complex is formed after 250 ns of simulation.

A similar characteristic that makes it possible to study the process of formation of the dendrimer–fullerene complex is the radius of gyration, *R*_g_ [112]

(1) $$R_{g}\left(t\right)=\sqrt{\frac{\sum_{i}^{N_{tot}}m_{i}{\left(r_{i}\left(t\right)\right)}^{2}}{\sum_{i}^{N_{tot}}m_{i}}}$$

where *m_i_* and *r_i_* are the mass index and radius vector of the *i*-th atom of the dendrimer–fullerene subsystem, and *N_tot_* is the total number of atoms in a dendrimer–fullerene complex.

The time dependence of the radius of gyration, *R*_g_(*t*), describes the process of equilibration of the size of the dendrimer–fullerene subsystem and the formation of their complex. Unlike *d*(*t*), the change in the value of *R*_g_(*t*) of this subsystem occurs not only due to a change in distance between dendrimer and fullerene but also due to fluctuations of the *R*_g_ of the dendrimer, which, unlike fullerene, is not a rigid molecule.

**Figure 2:**
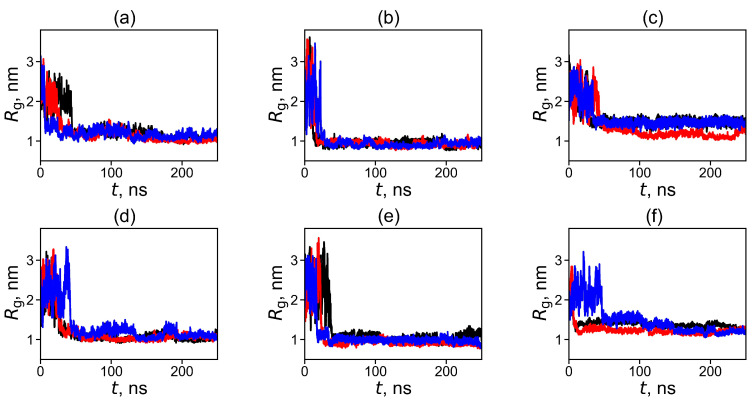
Radius of gyration (*R*_g_) of dendrimer and fullerene in the system: (**a**) Lys-2Gly + C_60_, (**b**) Lys G2 + C_60_, (**c**) Lys G3 + C_60_, (**d**) Lys-2Gly + C_70_, (**e**) Lys G2 + C_70_, (**f**) Lys G3 + C_70_ at the first 250 ns of a trajectory (before and after the formation of complex). For each system, the three different initial conformations (positions of fullerene) were used and marked by different colors. The abrupt decrease in the radius of gyration reflects the formation of dendrimer–fullerene complexes.

As can be seen in Figure 2, the subsystems have large initial radii of gyration *R*_g_(*t* = 0) since, at the beginning of the simulation, the fullerene is placed quite far from the center of mass of the dendrimer. The behavior of the *R*_g_(*t*) curves is similar to that of *d*(*t*) in Figure 1. The sizes of the resulting *R*_g_ of the complexes are about 1 nm. After the jump, the value of *R*_g_ decreases significantly and then remains practically unchanged with time. Thus, we can conclude that in all the systems under consideration the dendrimer forms a complex with fullerene.

### 2.2. Analysis of the Structure and Properties of Dendrimer–Fullerene Complexes Snapshots, Shapes, Sizes, and Distances between Dendrimer and Fullerene in Complexes

**Figure 3:**
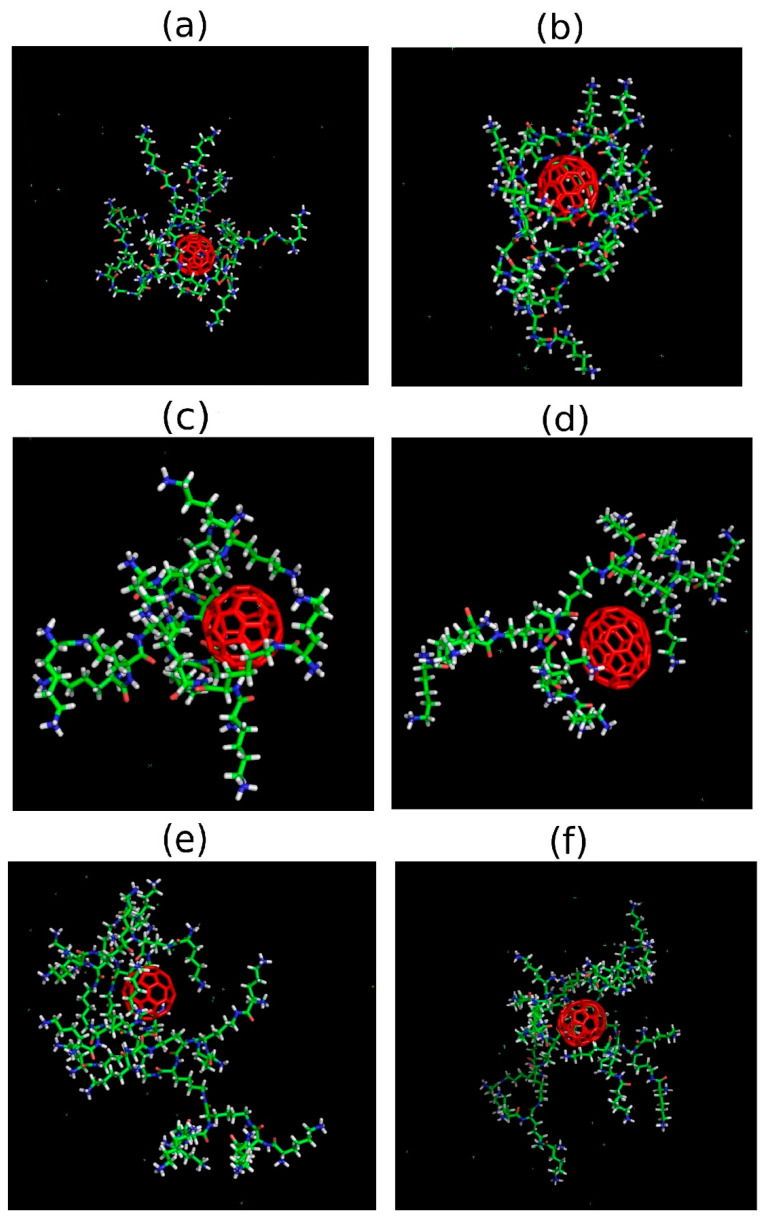
Snapshots of the dendrimer–fullerene system after 500 ns of simulation: (**a**) Lys-2Gly + C_60_, (**b**) Lys-2Gly + C_70_, (**c**) Lys G2 + C_60_, (**d**) Lys G2 + C_70_, (**e**) Lys G3 + C_60_, (**f**) Lys G3 + C_70_.

To demonstrate the relative position of the dendrimer and fullerene molecules in the complex, we present snapshots of these systems at the end of the simulation (after 500 ns) in Figure 3. It can be clearly seen that in all cases, the fullerene is located inside the dendrimer or, at least, between its branches. The additional visual analysis of snapshots confirms that after the first approach to the dendrimer, the fullerene molecule stays in contact with it all the time. This is in good agreement with the results presented in Figure 1 and Figure 2.

Based on the obtained trajectories, it is possible to calculate the equilibrium characteristics of the complexes and the dendrimer and fullerene molecules included in them (see Table 1).

To determine the shape of the complexes, we calculated the asphericity [113,114]:

(2) $$\alpha =1-\frac{3\left\langle I_{2}\right\rangle }{\left\langle I_{1}^{2}\right\rangle }$$

(3) $$I_{1}=R_{\mathrm{gx}}+R_{\mathrm{gy}}+R_{\mathrm{gz}}$$

(4) $$I_{2}=R_{\mathrm{gx}}R_{\mathrm{gy}}+R_{\mathrm{gy}}R_{\mathrm{gz}}+R_{\mathrm{gx}}R_{\mathrm{gz}}$$

The values of *α* = 0 and *α* = 1 correspond to the spherical and the elongated shape of the macromolecule, respectively. The values of asphericity are given in Table 1. Previously we established that for the dendrimers considered in this article, the asphericity of an individual dendrimer macromolecule is very close to zero in aqueous solution, i.e., the dendrimers in water have a shape close to spherical.

According to Table 1, for all complexes, except for Lys G3 + C_60_, the values of asphericity are smaller compared to that of dendrimer. However, even for Lys G3 + C_60_, *α* increases slightly, which means that the shape of Lys G3 + C_60_ complex remains close to spherical. This confirms that the sizes of the complexes can be described by one characteristic—the radius of gyration (*R*_g_). Also, to describe the structure of the resulting complexes, it will be sufficient to use the radial distribution functions described below.

**Figure 4:**
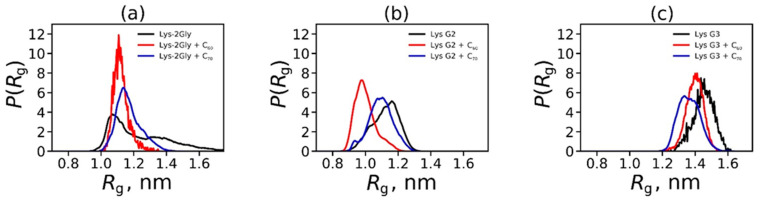
Distribution function for the radius of gyration (*R*_g_) of an individual dendrimer (in the absence of fullerene) and complexes of the dendrimer with C_60_ or C_70._ For Lys-2Gly (**a**), Lys G2 (**b**) and Lys G3 (**c**).

For all the studied complexes in the equilibrium state, the size (*R*_g_) of the dendrimer complex with fullerene is close to the size of an individual dendrimer (see Table 1). Moreover, for all complexes, *R*_g_, a decrease in the amplitude of *R*_g_ fluctuations is observed compared to the individual dendrimer. This happens because the hydrophobic fullerene tries to “hide” inside the dendrimer using the internal hydrophobic fragments of this macromolecule, which leads to the fullerene “gluing” the neighboring branches of the dendrimer and, as a result, decreases fluctuations and the average size of the complex. This is especially clearly visible if we consider the distribution function *P*(*R*_g_) over the radius of gyration *R*_g_ (Figure 4). As can be seen from this figure, the distribution of *P*(*R*_g_) for the complexes becomes narrower. This is especially noticeable for the complexes of Lys-2Gly with fullerenes.

To understand the degree of fullerene encapsulation, it is necessary to study the relative position of the dendrimer and fullerene molecules in the complex. For this purpose, the average distance from the center of mass of the dendrimer to the center of mass of the fullerene was calculated (see Table 1). It turned out that in the case of the Lys-2Gly + C_60_ and Lys-2Gly + C_70_ complexes, the distance between the centers of mass of the dendrimer and fullerene is the smallest (about 0.5–0.6 nm). This means very good penetration of the fullerene deep into the dendrimer, probably due to the greater length and flexibility of the glycine spacers between the branching points of the Lys-2Gly dendrimer. In the case of the Lys G2 and Lys G3 complexes with fullerenes, the distance between the centers of mass is slightly larger (about 0.7–0.8 nm) since these dendrimers have shorter and stiffer spacers. Therefore, in the latter case, a situation may arise when the fullerene is attached to the more external parts of the dendrimer branches. To obtain a deeper understanding of the internal structure of the complex, the next subsection examines the radial distribution functions of the atoms of the molecules in the complex.

### 2.3. Radial Distribution Functions

**Figure 5:**
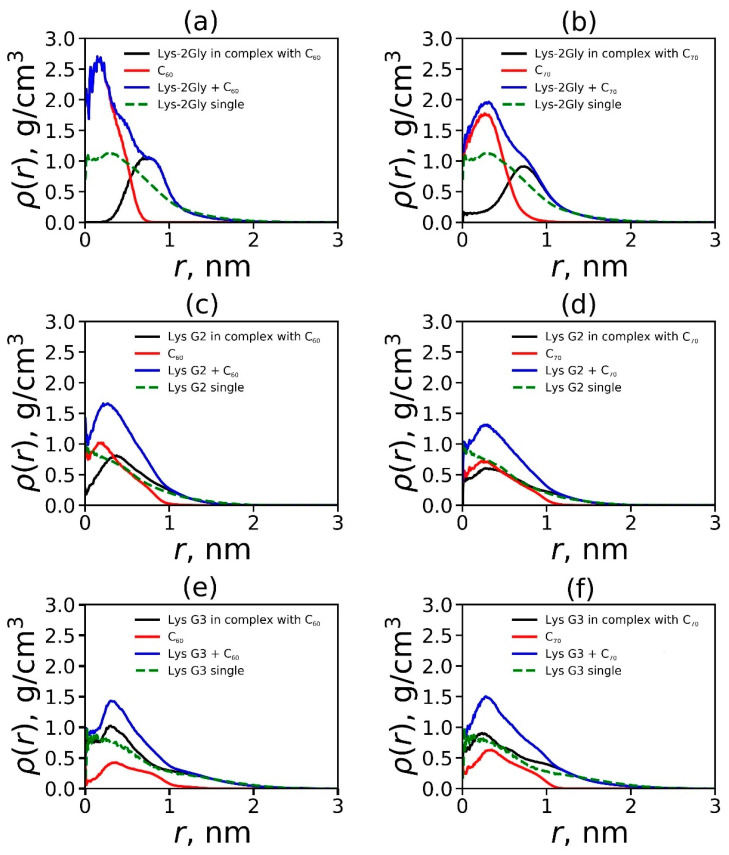
Density distribution for the dendrimer, fullerene, and the dendrimer–fullerene complex: (**a**) Lys-2Gly + C_60_ and (**b**) Lys-2Gly + C_70_, (**c**) Lys G2 + C_60_ and (**d**) Lys-G2 + C_70_, (**e**) Lys G3 + C_60_, and (**f**) Lys G3 + C_70_.

As shown in the previous subsection, the dendrimer–fullerene complex formed in all the considered systems. However, the degree of fullerene encapsulation may depend on the type of dendrimer and/or fullerene used. To obtain detailed information about the internal structure of the complex, the radial density distribution functions were calculated:

(5) $$\rho \left(r\right)=\frac{\langle m\left(r\right)\rangle }{V\left(r\right)}$$

where *ρ*(*r*) (in g/cm^3^) is the average density of the thin spherical layer at a distance *r* from the complex (or a dendrimer) center of mass, and $\langle m\left(r\right)\rangle$ is the average total mass of atoms in the same layer of volume *V*(*r*). The dependencies *ρ*(*r*) are presented in Figure 5.

It can be clearly seen that the relative positions of the dendrimer and fullerene in different complexes differ. In the case of Lys-2Gly (Figure 5a,b), the shape of the *ρ*(*r*) curves indicates that this dendrimer encapsulates fullerene quite well. In this case, the atoms of the Lys-2Gly dendrimer are displaced from the center of the mass region by fullerene and replaced by fullerene atoms. In the case of Lys G2 (Figure 5c,d, the displacement of the dendrimer atoms from the center of mass by fullerene atoms is also observed, but it is less pronounced. As for Lys G3 (Figure 5e,f), the maxima of *ρ*(*r*) curves for the dendrimer and fullerene are observed near the same distances from the center (dendrimer and fullerene atoms coexist there).

**Figure 6:**
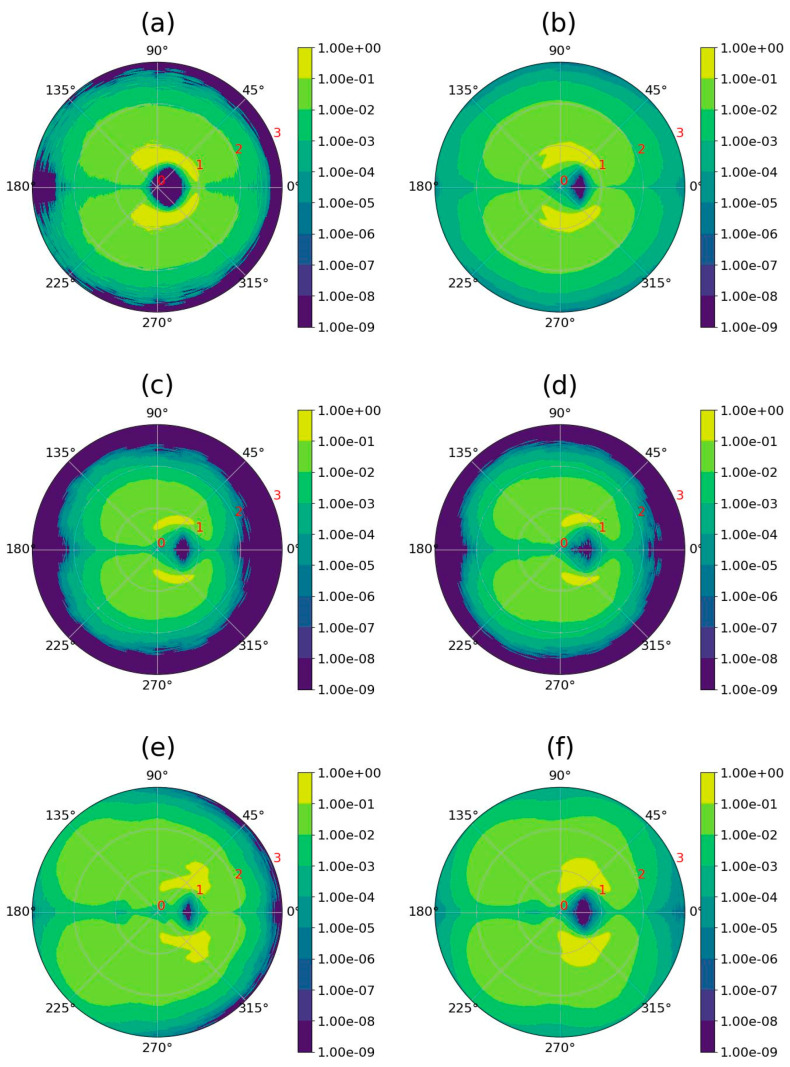
Two-dimensional sectoral radial distribution of mass of the dendrimer relative to its center of mass. The spherical sectors in which the functions were calculated using a vector directed from the center of mass of the dendrimer to the center of mass of the fullerene. The center of the sphere coincides with the center of mass of the dendrimer. The partition of a sphere into a set of spherical sectors is similar to the partition described in [115]. The difference is that we divided each sector into the upper (**left**) and lower (**right**) parts and calculated not only the angular distribution but also the radial distribution from the center of mass on each 2D plot. (**a**) Lys-2Gly + C_60_ and (**b**) Lys-2Gly + C_70_, (**c**) Lys G2 + C_60_ and (**d**) Lys-G2 + C_70_, (**e**) Lys G3 + C_60_, and (**f**) Lys G3 + C_70_.

To understand whether dendrimer–fullerene interactions depend only on the radial distance *r* or whether there is an additional angular dependence [115], we plotted the two-dimensional sectoral radial mass distributions relative to the center of mass of the dendrimer (Figure 6).

In Figure 6, the area of the reduced dendrimer density (dark blue), close to the center of the figure, corresponds to the position of the fullerene. It can be seen that for all systems, the dendrimer atoms are located constantly (yellow area), often (yellow-green area), and less often (green area) around the fullerene. It is important to note that the largest number of dendrimer atoms (yellow region) are located near the fullerene and form a region of constant contact with it but do not form a closed shell around it. However, regions with fewer atoms (yellow-green and green), located on average at a slightly greater distance from it, completely surround the fullerene and protect it on all sides (at least partially) from contact with water. To summarize this part of the work, it should be noted that the encapsulation of fullerene with the Lys-2Gly dendrimer is more effective compared to other studied lysine dendrimers (Lys G2 and Lys G3). Apparently, this may be due to the longer and more flexible glycine spacers of this dendrimer, in contrast to the shorter and more rigid spacers of the other two lysine dendrimers.

### 2.4. Electrostatic Properties of the Dendrimer–Fullerene Complex and Individual Dendrimer

One of the most promising applications of dendrimers is encapsulation and delivery of drugs. As shown above, the dimensions of the dendrimer practically do not change after encapsulation of fullerene.

**Figure 7:**
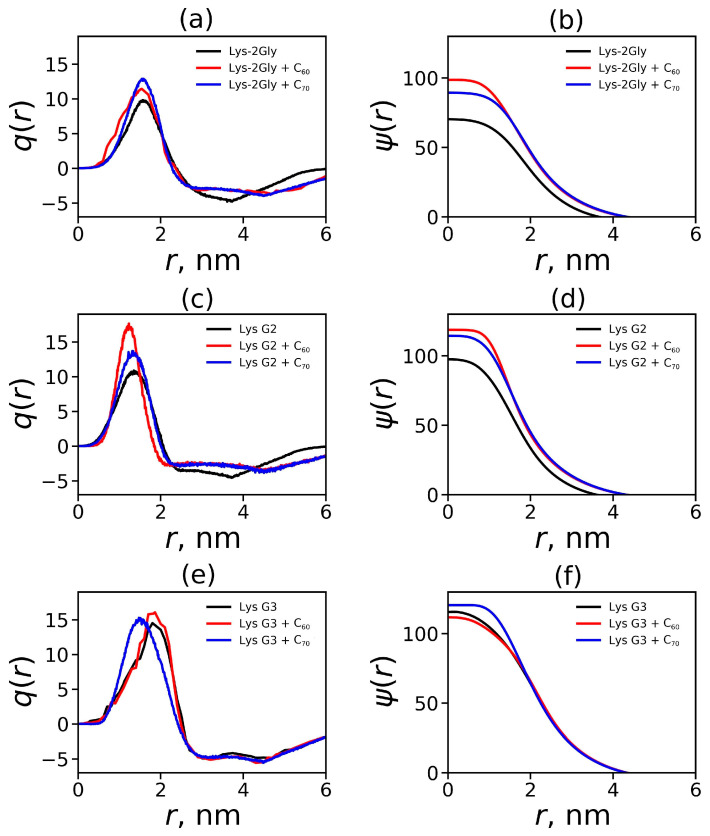
Electrostatic characteristics of the dendrimers and their complexes with fullerenes: the radial charge distribution, *q*(*r*), (**a**,**c**,**e**) and electrostatic potential, *ψ*(*r*), (**b**,**d**,**f**) for Lys-2Gly (**a**,**b**), Lys G2 (**c**,**d**), and Lys G3 (**e**,**f**).

In this section, we studied how the encapsulation of fullerene affects the electrostatic characteristics of the dendrimer–fullerene complex compared to those of an individual dendrimer in an aqueous solution. For this purpose, the radial charge distribution of the dendrimer and counterions, *q*(*r*), was calculated. Then, using *q*(*r*), we solved the Poisson equation numerically and obtained the distribution of the electrostatic potential, *ψ*(*r*) [116,117,118] (Figure 7). The averaged electrostatic characteristics and the number of hydrogen bonds between the dendrimer and water are given in Table 2. In Figure 7a,c, for the complexes of Lys-2Gly and Lys G2 with fullerenes, we can observe an increase in the height of the positive peak of *q*(*r*) compared to that for the separate dendrimers while the negative peak of *q*(*r*) is smaller and wider. The electrostatic potential for these two complexes is also stronger for these complexes compared to single dendrimers, especially at low values of *r*. At the same time, the formation of the complexes of Lys G3 with fullerenes leads to less pronounced changes in the electrostatic parameters compared to those for the separate dendrimer (Figure 7e,f), especially at higher distances r from the center of mass of this dendrimer.

In the case of Lys-2Gly and Lys G2, a change in the charge distribution and electrostatic potential of the dendrimer–fullerene complexes leads to a slight increase in the maximal cumulative charge, *Q*_max_ (see Table 2). At the same time, a significant increase (by 1.5–1.7 times) in the ζ potential of these complexes compared to these parameters for individual dendrimers occurs. As for Lys G3, the ζ potential of its complexes with fullerenes C_60_ and C_70_ increase slightly (by 1.07 and 1.13 times, correspondingly). The number of hydrogen bonds between all dendrimer–fullerene complexes and water molecules was different but approximately the same as between the corresponding dendrimer and water (see Table 2).

## 3. Materials and Methods

**Figure 8:**
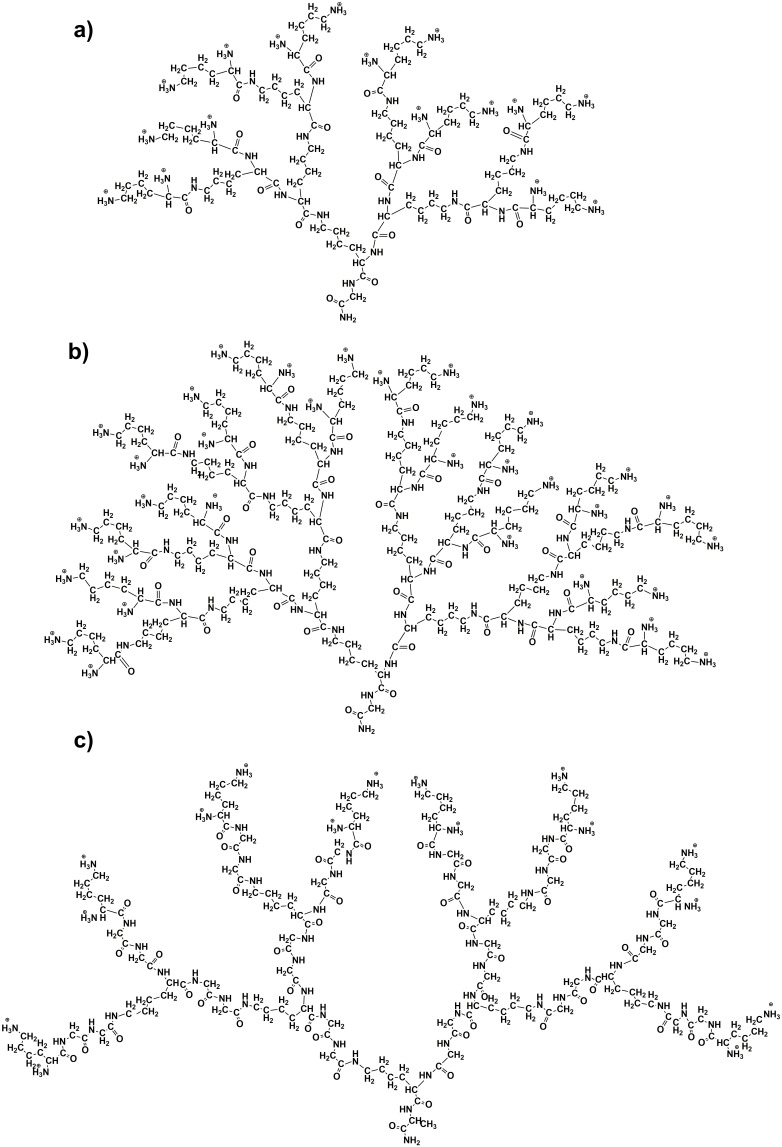
The chemical structure of (**a**) the lysine dendrimer of the second generation, Lys G2, and (**b**) the lysine dendrimer of the third generation, Lys G3; (**c**) the second-generation lysine-based dendrimer with double glycine (2Gly) insertions between adjacent branching points, Lys-2Gly.

In this work, we studied the possibility of the formation of complexes of lysine-based peptide dendrimers with fullerenes in an aqueous solution using the molecular dynamics (MD) method. The MD simulations were carried out using the GROMACS package [119]. AMBER-99SB-ILDN was used as the force field [120]. The lysine dendrimers of the second (Lys G2) and third (Lys G3) generations and the lysine-based dendrimer (Lys-2Gly) with double insertions of glycine amino acid residues (2Gly) between neighboring branching points of Lys G2 dendrimer were considered (Figure 8). We used the full atomic models of these dendrimers. Table 3 contains some structural parameters of the dendrimers under consideration.

The initial conformations of the dendrimers were taken from previous work [121]. The topologies and partial charges of fullerenes C_60_ and C_70_ were prepared using an electronic resource [122]. At the initial moment of time, the fullerene molecule was placed at a certain distance from the dendrimer. For each of the six systems (three dendrimers and two types of fullerenes), three different initial conformations of the system (positions of fullerene) were used. For this, we shifted the center of mass of the fullerene relative to the center of mass of the dendrimer along one of three different axes, X, Y, or Z, by the same distance of the order of 4.5 nm so that the fullerene in all cases was outside the dendrimer surface but not too far from it. The preparation for the simulation included the following stages: First, the corresponding dendrimer and fullerene molecules were immersed in an aqueous solution. According to normal pH, the terminal groups of the dendrimer were protonated. Then, chlorine counterions were added to satisfy the electrical neutrality condition of the simulated systems.

The final equilibration was carried out for 250 ns in an NPT ensemble using a Nose–Hoover thermostat and a Parrinello–Rahman barostat. The simulation parameters were exactly the same as in our previous works [121]. After equilibration, the simulation was continued, and the length of the productive trajectory was equal to 250 ns. This trajectory was used to calculate different average values, distribution functions, and time-dependent properties at equilibrium using both GROMACS tools and developed computer programs for linear polymers [123,124,125,126,127,128], dendrimers [129,130,131,132,133].

## 4. Conclusions

In this work, atomistic molecular dynamics simulations were used for the first time to study the possibility of the complexation of fullerenes with amphiphilic peptide dendrimers. Six systems were considered, containing one of three peptide dendrimers (Lys-2Gly, Lys G2, and Lys G3) and one of two types of fullerenes (C_60_ and C_70_) in an aqueous solution. It was shown that in all six considered systems, a dendrimer–fullerene complex is formed. In addition, it turned out that fullerene can be encapsulated quite close to the center of the dendrimer. In most of the complexes considered, the density and hydrophobicity of the internal region of the complex are greater than that of a single dendrimer, which makes it less accessible to water and counterions. This reduces the number of counterions inside the complex and increases its zeta potential. At the same time, the fullerene “glues” the branches of the dendrimer, which reduces fluctuations and the overall dimensions of the complex. It was found that in all the complexes considered, the dendrimer surrounds the fullerene molecule with its branches, hiding it from water. It was demonstrated that the dendrimer Lys-2Gly exhibits the best encapsulating properties for both types of fullerenes. It is concluded that all the considered peptide dendrimers and especially the Lys-2Gly dendrimer can be good nanocontainers for the delivery of fullerenes.

## Figures and Tables

**Table 1:** The asphericity *α*, the radius of gyration *R*_g_ of the dendrimer, and the dendrimer–fullerene complex, as well as mean square distance *d* between the centers of mass of the fullerene and dendrimer.

Dendrimer/Fullertene	*α*	*R*_g_	*d*
Lys-2Gly	0.013	1.24	-
Lys-2Gly + C_60_	0.006	1.04	0.51
Lys-2Gly + C_70_	0.006	1.08	0.57
Lys G2	0.021	1.12	-
Lys G2 + C_60_	0.009	0.91	0.72
Lys G2 + C_70_	0.018	0.98	0.77
Lys G3	0.015	1.45	-
Lys G3 + C_60_	0.018	1.46	0.81
Lys G3 + C_70_	0.012	1.27	0.78

**Table 2:** Electrostatic properties: the maximal cumulative charge, *Q*_max_, and the ζ potential; the number of hydrogen bonds (*N*_HB_) of the dendrimer/dendrimer–fullerene complex with water.

System	*Q*_max_ [e]	ζ [mV]	*N*_HB_
Lys-2Gly	9.3	21.2	101
Lys-2Gly + C_60_	11.3	35.1	96
Lys-2Gly + C_70_	11.3	35.2	100
Lys G2	10.1	30.9	56
Lys G2 + C_60_	11.5	52.8	55
Lys G2 + C_70_	11.4	45.1	56
Lys G3	15.7	37.4	107
Lys G3 + C_60_	15.8	40.1	105
Lys G3 + C_70_	15.5	42.3	104

**Table 3:** Characteristics of dendrimers: the molecular mass of the dendrimer (*M*_d_), the charge (*Q*_bare_), the number of positively charged terminal NH_3_^+^ groups (*N*_end_), the total number of amino acid residues in the insertions (*N*_ins_), the total number of amino acid residues in the dendrimer (*N*_res_).

Dendrimer	*M*_d_, g/mol	*Q*_bare_ [e]	*N*_end_	*N*_ins_	*N*_res_
Lys-2Gly	3675.44	16	16	28	44
Lys G2	1895.63	16	16	0	16
Lys G3	3962.5	32	32	0	32

## Data Availability

Data is contained within the article.
